# SUR1-TRPM4 channel activation and phasic secretion of MMP-9 induced by tPA in brain endothelial cells

**DOI:** 10.1371/journal.pone.0195526

**Published:** 2018-04-04

**Authors:** Volodymyr Gerzanich, Min Seong Kwon, Seung Kyoon Woo, Alexander Ivanov, J. Marc Simard

**Affiliations:** 1 Department of Neurosurgery, University of Maryland School of Medicine, Baltimore, Maryland, United States of America; 2 Department of Pathology, University of Maryland School of Medicine, Baltimore, Maryland, United States of America; 3 Department of Physiology, University of Maryland School of Medicine, Baltimore, Maryland, United States of America; Indiana University School of Medicine, UNITED STATES

## Abstract

**Background:**

Hemorrhagic transformation is a major complication of ischemic stroke, is linked to matrix metalloproteinase-9 (MMP-9), and is exacerbated by tissue plasminogen activator (tPA). Cerebral ischemia/reperfusion is characterized by SUR1-TRPM4 (sulfonylurea receptor 1—transient receptor potential melastatin 4) channel upregulation in microvascular endothelium. In humans and rodents with cerebral ischemia/reperfusion (I/R), the SUR1 antagonist, glibenclamide, reduces hemorrhagic transformation and plasma MMP-9, but the mechanism is unknown. We hypothesized that tPA induces protease activated receptor 1 (PAR1)-mediated, Ca^2+^-dependent phasic secretion of MMP-9 from activated brain endothelium, and that SUR1-TRPM4 is required for this process.

**Methods:**

Cerebral I/R, of 2 and 4 hours duration, respectively, was obtained using conventional middle cerebral artery occlusion. Immunolabeling was used to quantify p65 nuclear translocation. Murine and human brain endothelial cells (BEC) were studied *in vitro*, without and with NF-κB activation, using immunoblot, zymography and ELISA, patch clamp electrophysiology, and calcium imaging. Genetic and pharmacological manipulations were used to identify signaling pathways.

**Results:**

Cerebral I/R caused prominent nuclear translocation of p65 in microvascular endothelium. NF-κB-activation of BEC caused *de novo* expression of SUR1-TRPM4 channels. In NF-κB-activated BEC: (i) tPA caused opening of SUR1-TRPM4 channels in a plasmin-, PAR1-, TRPC3- and Ca^2+^-dependent manner; (ii) tPA caused PAR1-dependent secretion of MMP-9; (iii) tonic secretion of MMP-9 by activated BEC was not influenced by SUR1 inhibition; (iv) phasic secretion of MMP-9 induced by tPA or the PAR1-agonist, TFLLR, required functional SUR1-TRPM4 channels, with inhibition of SUR1 decreasing tPA-induced MMP-9 secretion.

**Conclusions:**

tPA induces PAR1-mediated, SUR1-TRPM4-dependent, phasic secretion of MMP-9 from activated brain endothelium.

## Introduction

Low concentrations of tissue plasminogen activator (tPA) are neuroprotective [1], and thrombolytic therapy employing recombinant tPA (rtPA) greatly improves stroke outcome [2]. However, in cerebral ischemia, the administration of recombinant rtPA for thrombolysis can exacerbate breakdown of the blood-brain barrier (BBB), worsen brain edema, and increase the incidence and severity of hemorrhagic transformation [3]. The vascular toxicity of rtPA, which is tied to matrix metalloproteinases (MMP), has prompted the development of numerous strategies targeting various molecular pathways linked to MMP, most of which are plasminogen/plasmin-independent [4,5]. However, plasmin, the principal downstream product of tPA, also may cause opening of the BBB [5]. To date, however, the involvement of a plasmin-linked mechanism in MMP secretion by endothelium has not been extensively studied.

Plasmin is a serine protease that can induce canonical activation of protease-activated receptor 1 (PAR1) by cleaving PAR1 at Arg41 [6]. In ischemia, PAR1 is upregulated by cerebral endothelium [7]. Canonical PAR1 activation leads to classical G-protein coupled receptor (GPCR) signaling, including in endothelium [8,9]. GPCR signaling involves the hydrolysis of phosphatidylinositol 4,5-bisphosphate (PIP_2_), generation of diacylglycerol (DAG), and activation DAG-sensitive transient receptor potential canonical (TRPC) 3/6 channels, which mediate sustained Ca^2+^ influx.

In humans with stroke and in rat models of stroke, SUR1-TRPM4 (sulfonylurea receptor 1 –transient receptor potential melastatin 4) channels [10] are upregulated in all members of the neurovascular unit [11,12], with microvascular endothelial cells being the earliest cell-type to upregulate the channel [13]. SUR1-TRPM4 channels, which conduct monovalent but not divalent cations [14,15], are opened by intracellular Ca^2+^, which acts via calmodulin and a calmodulin binding site at the C terminus of TRPM4 [16]. SUR1-TRPM4 channel opening results in Na^+^ influx [14,15]. Cell depolarization due to Na^+^ influx reduces the inward electrochemical driving force for Ca^2+^, thus acting as a negative regulator to reduce Ca^2+^ influx [17] by channels such as TRPC3 [18]. These characteristics of SUR1-TRPM4 raised the possibility that SUR1-TRPM4 could be involved in PAR1 signaling linked to Ca^2+^ influx in endothelium.

In humans with stroke and in rodent models of stroke, the SUR1 inhibitor, glibenclamide, has been found to reduce hemorrhagic transformation and plasma levels of MMP-9 [19–22], but the mechanism is unknown. ProMMPs, which require proteolytic cleavage to become mature proteases, are secreted via two distinct mechanisms: (i) MMPs undergo *tonic* secretion via the normal secretory pathway [23]; (ii) MMPs undergo *phasic* secretion following GPCR activation [24], the latter requiring Ca^2+^ influx [25]. Here, we hypothesized that tPA induces PAR1-mediated, Ca^2+^-dependent phasic secretion of MMP-9 from activated brain endothelium, and that SUR1-TRPM4 channels are required for this process.

## Materials and methods

### Ethics statement, and care and use of animals

We certify that all applicable institutional and governmental regulations concerning the ethical use of animals were followed during the course of this research. Animal experiments were performed under a protocol approved by the Institutional Animal Care and Use Committee of the University of Maryland, Baltimore and in accordance with the relevant guidelines and regulations as stipulated in the United States National Institutes of Health Guide for the Care and Use of Laboratory Animals. The University of Maryland School of Medicine Veterinary Resources Program is fully accredited by the American Association for the Accreditation of Laboratory Animal Care. The program of animal care is directed by full-time specialty-trained, laboratory animal veterinarians. All efforts were made to minimize the number of animals used and their suffering.

### Cerebral ischemia/reperfusion

Methods used in this laboratory for cerebral ischemia/reperfusion (I/R), i.e., temporary middle cerebral artery occlusion (MCAo), have been detailed [26,27]. Briefly, male Wistar rats (275–325 g, Harlan, Indianapolis, IN) were anesthetized (ketamine 60 mg/kg and xylazine 7.5 mg/kg intraperitoneally). SpO_2_ via pulse oximetry and temperature were carefully regulated, and all surgical procedures were performed aseptically. MCAo was obtained using a commercially available intra-arterial occluder (0.39 mm; 4039PK5Re; Doccol Corp, Redlands CA) inserted retrograde into the external carotid artery and advanced into the internal carotid artery under guidance of a laser Doppler flowmeter. Only animals with a drop in relative cerebral blood flow >75% were included for further study. After 120 minutes of MCAo, the occluder was withdrawn to allow reperfusion. The animals were euthanized (sodium pentobarbital, ≥100 mg/kg, followed by transcardial exsanguination) after 4 hours of reperfusion, i.e., 6 hours after onset of I/R, and the brains were analyzed by immunohistochemistry.

### Immunohistochemistry

Coronal cryosections (12 μm) were blocked for 1 hour in 2% donkey serum and 0.2% TritonX-100. Sections were incubated overnight at 4 °C with primary antibody against p65 (GTX102090; Genetex, Irvine CA) and double-labeled with primary antibody against rat endothelial cell antigen-1 (RECA-1) (MA1-81510; Thermo Fisher Scientific Inc.). Species appropriate Alexa Fluor 555- or fluorescein isothiocyanate-conjugated secondary antibodies were used for visualization. Sections were coverslipped with ProLong Gold antifade reagent (P36930; Thermo Fisher Scientific Inc.) containing the nuclear stain, DAPI (4′,6-Diamidine-2′-phenylindole dihydrochloride). Omission of primary antibody was used as a negative control.

Unbiased measurements of signal intensity within regions of interest (ROIs) were obtained using NIS-Elements AR software (Nikon Instruments, Melville, NY, USA), as described previously [28]. The area that was evaluated was a rectangle, 360 μm × 440 μm, positioned within the ACA-MCA watershed territory. Specific labeling for p65 within the ROI was defined as pixels with signal intensity >2.0× background. For nuclear translocation of p65, the ROI was defined by DAPI labeling within the same territory.

### Reagents for *in vitro* experiments

Reagents (used at the concentrations indicated), were obtained from: Calbiochem (San Diego, CA): MMP-9 and MMP-2, recombinant (25 ng/mL); Genetech (San Francisco, CA): tPA (Cathflo^®^ Activase^®^ [Alteplase]; 20 μg/mL); Santa Cruz Biotechnology Inc. (Santa Cruz, CA): MMP inhibitor II (300 nM); siRNA targeting TRPM4; Sigma (St. Louis, MO): 9-phenanthrol (100 μg/mL); A23187 (5 μM); aprotinin (10 μg/mL); diazoxide (100 μM); Gd^+3^ (100 μM); glibenclamide (3 μM unless otherwise noted; #G2539); glutamate plus glycine (1 mM and 20 μM, respectively); L-arginine (700 μg/mL); leupeptin (10 μg/mL); plasmin (1 μg/mL); pyrrolidine dithiocarbamate (PDTC; 100 μM); Pyr3 (10 μM); ruthenium red (10 μM); SKF-96365 (10 μM); sodium azide plus 2-deoxyglucose (to deplete ATP; 1 mM plus 10 mM, respectively); TFLLR (30 μM); TNF (1–20 ng/mL); tranexamic acid (100 μM); Tocris Bioscience (Bristol UK): RWJ56110 (5 μM); SFLLRN (TRAP-6) (10 μM).

### Cell culture

Immortalized murine BEC (bEnd.3; ATCC, Gaithersburg, MD) or immortalized human BEC (Lonza, Basel, Switzerland) were cultured as described [13]. For some experiments, BEC were isolated from wild-type or *Abcc8*–/–male mice (25–30 gm) using a modification of the method described [29]. Endothelial cell activation was obtained by exposure to TNF (20 ng/mL) for 3–16 hours under serum-free conditions.

### Luciferase promoter assay

TNF-stimulated activity of the promoter region of *Abcc8* and *Trpm4* was determined using luciferase reporter plasmids, as previously described [13,30]. Photinus luciferase reporter plasmids containing the rat *Abcc8* promoter (–578 to +80), or mouse *Trpm4* promoter (–1867 to +105), or four consecutive NF-κB consensus sequences (Clonetech, Mountain View, CA) were transfected into HepG2 cells using Lipofectamine 2000 (Invitrogen). Co-transfection of pRL-CMV, Renilla luciferase expression plasmid served as a control for transfection efficiency. After transfection, the cells were maintained in basal conditions for 24 hours, then exposed to TNF (20 ng/mL) or vehicle for another 24 hours. The activities of the Photinus and Renilla luciferases in extracts of the transfected cells were measured using the Dual Luciferase Reporter Assay System (Promega, Madison, WI). The Photinus luciferase activity was divided by the Renilla activity from the same sample to normalize the transfection efficiency. The divided values were then expressed relative to those from cells transfected with chicken β-actin promoter-driven luciferase in each condition. To obtain the fold-induction of luciferase activity by TNF, the values with TNF were divided by those from corresponding control samples or empty vector transfection [31].

### Immunoblot

Immunoblots were performed as described [10], using rabbit anti-CaMKII (pan; #3362S) or rabbit anti-pThr286-CaMKII (D21E4; #12716S), mouse anti-ERK1/2 (3A7; #9107), rabbit anti-pThr202/Tyr204-ERK1/2 (197G2; #4377), all from Cell Signaling Technology, Danverse MA.

### PCR

RT-PCR and qRT-PCR were carried out as described [11] using the primers listed in Tables 1 and 2.

**Table 1:** Primer sequences for RT-PCR.

Gene	Ref Seq	Forward	Reverse
β-actin	NM_007393	CAACTGGGACGACATGGAGAA	CAGCCTGGATGGCTACGTACA
MMP9	NM_013599	TTGAGTCCGGCAGACAATCC	CCTTATCCACGCGAATGACG
Abcc8	NM_011510	CCCTCTACCAGCACACCAAT	CTGATGCAGCACCGAAGATA
Trpm4	NM_175130	GCTTTCGTGCCCCAAACTTG	AGACCGTCAGCAACTCCTTTCG

**Table 2:** Primer sequences for qRT-PCR.

Gene	Ref Seq	Forward	Reverse
MMP2	NM_008610	CAAGGATGGACTCCTGGCACAT	TACTCGCCATCAGCGTTCCCAT
MMP9	NM_013599	GCTGACTACGATAAGGACGGCA	TAGTGGTGCAGGCAGAGTAGGA

### Patch clamp

Patch clamp electrophysiology was performed as described [10]. For whole cell recordings, we used a nystatin perforated patch technique. Nystatin, 50 mg (Calbiochem) was dissolved in 1 mL dimethylsulfoxide (DMSO). Working solutions were made before the experiment by adding 16.5 μL nystatin stock solution to 5 mL of the base pipette solution to yield a final concentration of nystatin of 165 μg/mL and DMSO 3.3 μl/mL.

To record whole cell macroscopic currents under “physiological” conditions, the extracellular solution contained (mM): NaCl 130, KCl 10, CaCl_2_ 1, MgCl_2_ 1, HEPES 32.5, glucose 12.5, pH 7.4; and the pipette solution contained (mM): KCl 55, K_2_SO_4_ 75, MgCl_2_ 8, and HEPES 10, and nystatin, 165 μg/mL, pH 7.2.

To record whole cell macroscopic currents exclusive of K^+^ channels, the extracellular solution contained (mM): CsCl 145, CaCl_2_ 1, MgCl_2_ 1, HEPES 32.5, glucose 12.5, pH 7.4; and the pipette solution contained (mM): CsCl 145, MgCl_2_ 8, and HEPES 10, and nystatin, 165 μg/mL, pH 7.2.

For inside-out patch recording, we used a bath solution containing (mM): CsCl 145, MgCl_2_ 1, CaCl_2_ 0.2, EGTA 5, HEPES 10, pH 7.3. The pipette contained (mM): CsCl 145, CaCl_2_ 1.5, MgCl_2_ 1, EGTA 5, HEPES 32.5, glucose 12.5, pH 7.4.

For cell-attached patch recording, both the pipette and the bath contained (mM): CsCl 145, CaCl_2_ 1.5, MgCl_2_ 1, HEPES 32.5, glucose 12.5, pH 7.4.

The following voltage clamp parameters were used: holding potential, –50 mV; ramp pulses were from –100 to +100 mV, 4 mV/msec, applied every 15 seconds; step pulses were 300 msec pulses from –100 mV to + 100 mV in 20-mV steps, applied every 2000 msec. Steady-state inward currents were quantified at –50 mV, and are presented in bar graphs as positive values.

### Calcium measurements

bEnd.3 cells grown on Fluorodish (WPI, Sarasota, FL) were activated with 20 ng/mL TNF for 3–16 hours. The cells were rinsed twice with a bath solution (HBSS containing 10 mM HEPES, pH 7.4) and then incubated with 5 μM Fluo-4 AM and 0.02% F-127 pluronic acid in the bath solution in the dark for 1 hour at room temperature [32,33]. The cells were washed twice with the bath solution and mounted in an inverted microscope. Changes in cytoplasmic free [Ca^2+^] in individual cells were measured by using a Zeiss LSM 510 confocal microscope with an argon laser (Zeiss, Jena, Germany) with excitation at 488 nm and emission at 515–530 nm. Fluorescence images (512 × 512 pixels) were obtained at 4-second intervals. To quantify fluorescence, pixel intensities within the selected single cell areas of interest were processed with the LSM Image Examiner software (Zeiss). Data are reported as mean change in [Ca^2+^] at 10–12 minutes following drug application.

### Gelatin zymography

Zymography was performed to measure the enzymatic activities of matrix metalloproteases (MMPs) from BEC culture media. The media under various conditions were collected and concentrated up to 75-fold using a commercial ultrafiltration device with a 10 kDa nominal molecular weight limit (EMD Millipore, Billerica, MA). The concentrated samples were separated on a Novex 10% zymogram protein gel containing 0.1% gelatin (Life Technologies, Grand Island, NY). The gelatin degrading proteolytic activities of the MMPs on the gel were detected by following the protocols provided by the manufacturer. Briefly, after electrophoresis, the gel was incubated with a renaturing buffer for 30 minutes at room temperature and with a developing buffer for 24–96 hours, depending on the amount of enzyme in the samples. To detect MMP activities in the gel, the gel was stained by a Coomassie G-250 solution (Life Technologies).

### Silencing *Abcc8*

A control lentiviral vector expressing EGFP under the control of CMV promoter, pSMPUW-CMV-EGFP was obtained from the Recombinant Virus Core Facility, University of Maryland, Baltimore. To construct a lentiviral vector expressing mouse *Abcc8*-specific shRNA with EGFP, pSMPUW-CMV-EGFP-mi-shAbcc8, we amplified ~250 bp-long DNA fragment containing miR-30 based shAbcc8 cassette using the pLenti-GFAP-EGFP-mi-shAct1 (kind gift of Dr. Zhang, Department of Neurology, Thomas Jefferson University, Philadelphia, PA) as a template and cloned downstream of EGFP in pSMPUW-CMV-EGFP by recombinant PCR and standard molecular cloning methods. All plasmids involved in PCR were validated by sequencing. The shRNA sequences in the miR-30 based shAbcc8 cassette used in this study include mouse shAbcc8-a (5’-TCCCGCCACCTCCATTTGTACATGCCAGC-3’) and mouse shAbcc8-b (5’-TTCGAGAGTCCCTTCAATAAGCAAAGGTA-3’). We used a commercial service for lentivirus packaging and production with a titer of ~1x10^6^ IU/mL, available at the Recombinant Virus Core Facility, University of Maryland.

To inhibit expression of *Abcc8*, 3x10^4^ bEnd.3 cells or mouse insulinoma cells (β-TC-6; ATCC, Gaithersburg, MD), which were used as a positive control, were transduced with the 1×10^5^ shAbcc8 lentivirus for 4 days and cultured for 3 additional days with daily replacement of the culture medium.

### Silencing *Trpm4*

Control and *Trpm4* siRNA (Santa Cruz Biotechnology) was transfected into bEnd.3 cells using the Cell Line Nucleofector Kit (Lonza; Walkersville, MD).

### Data analysis

Data are presented as mean±S.E. Statistical analyses were performed using Origin Pro (V8; OriginLab Corp., North Hampton MA). Student’s t-test or 1-way ANOVA with Fisher’s post-hoc comparisons were used for statistical evaluations.

## Results

**Fig 1:**
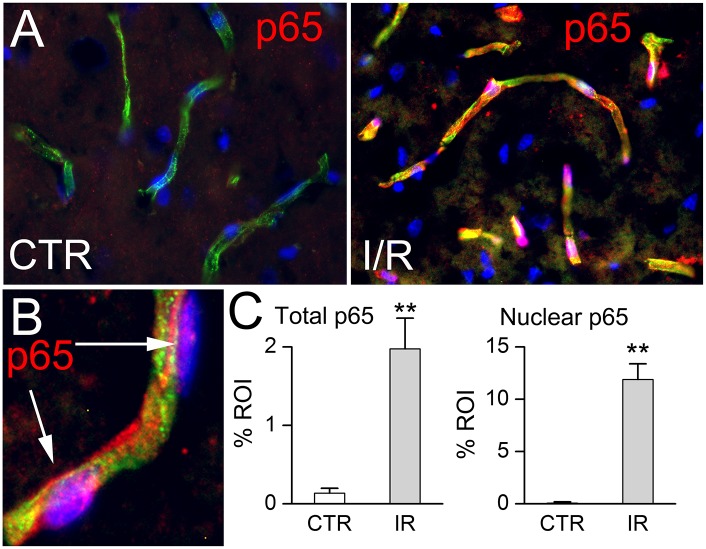
NF-κB activation *in vivo* after cerebral ischemia / reperfusion. **A**: Immunolabeling for p65 (red) in coronal sections from the middle cerebral artery territory of a control rat (*left*) and 6 hours after onset of I/R (*right*), showing nuclear translocation of p65 following I/R, but not in control; tissues were double immunolabeled for the endothelial marker, RECA-1 (green), and nuclei were stained with DAPI (4′,6-diamidine-2′-phenylindole dihydrochloride; blue); p65 (red) within nuclei (blue) of microvascular endothelial cells appears pink. **B**: At high magnification, nuclear p65 following I/R appears pink. **C**: Bar graphs quantifying increases in total p65 (*left*) and in nuclear p65 (*right*) following I/R; n = 5 rats/condition; **, *P*<0.01.

One of the hallmarks of ischemia/reperfusion (I/R) of the brain is activation of NF-κB [34–36]. In the rat MCAo model, 2 hours of ischemia followed by 4 hours of reperfusion resulted in robust NF-κB activation, most prominently in microvessels (Fig 1A). At 6 hours, there was a significant increase in total p65 as well as nuclear p65 (Fig 1B and 1C). Cerebral I/R also is well established to cause upregulation of MMP-9 [37,38] and of SUR1-TRPM4 [11,13] in microvascular endothelium. We thus elected to study the relationship between NF-κB activation, MMP-9 and SUR1-TRPM4 in endothelial cell cultures.

**Fig 2:**
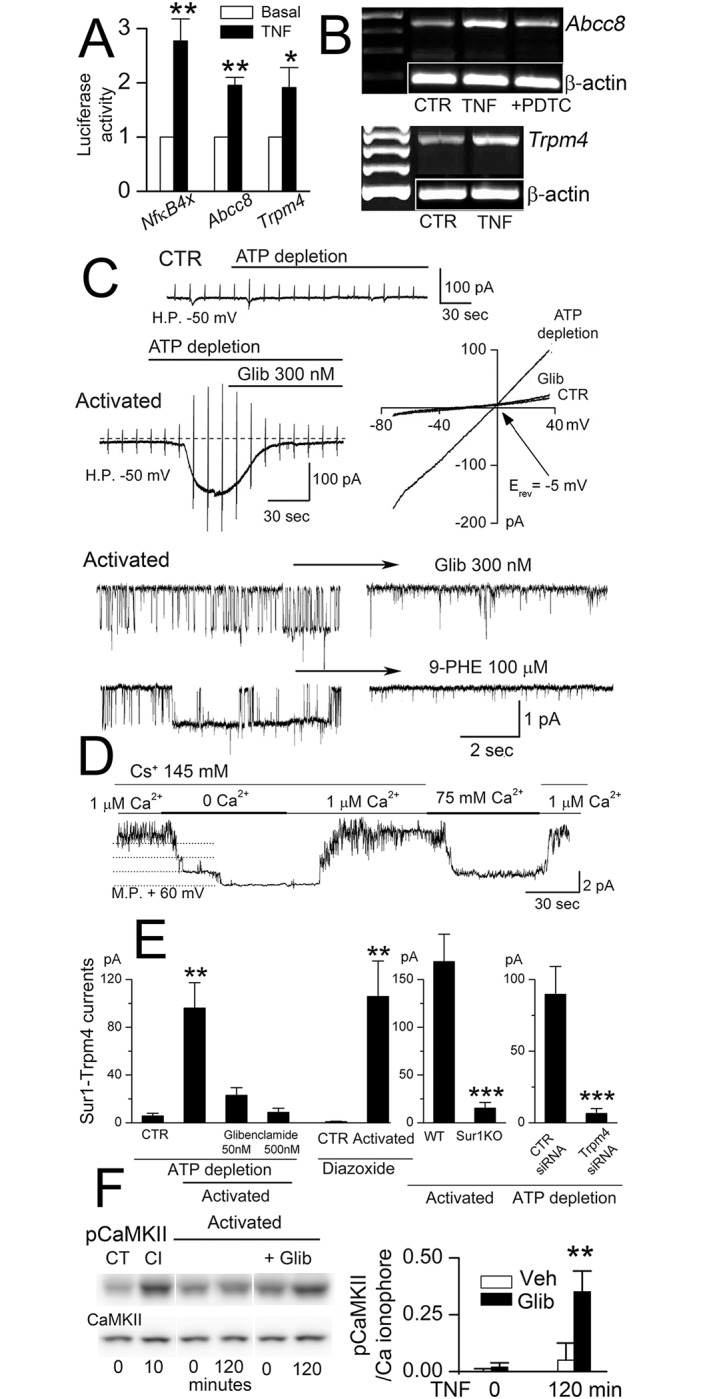
SUR1-TRPM4 channel upregulation *in vitro*. **A**: Activity of the *Abcc8* promoter, the *Trpm4* promoter and a positive control (four consecutive NF-κB consensus sequences), under basal conditions and after stimulation by TNF (20 ng/mL); 3 replicates; *, *P*<0.05; **, *P*<0.01. **B**: Murine BEC were exposed to TNF (20 ng/mL) and lysate was analyzed by RT-PCR 16 hours later; transcripts for *Abcc8* and *Trpm4* were upregulated compared to control (CTR); the induction of *Abcc8* mRNA by TNF was reduced by the NF-κB inhibitor, PDTC (pyrrolidinedithiocarbamate); β-actin mRNA was used as a loading control; representative of 3 replicates. **C**: Macroscopic and single channel (inside-out patch) currents induced by ATP depletion in activated but not in non-activated (CTR) murine BEC; currents were blocked by glibenclamide (Glib) and 9-phenanthrol (9-PHE). **D**: Single channel current in an inside-out patch with 4 single channel levels during changes in bath solution from 145 mM Cs^+^/1 μM Ca^2+^, 145 mM Cs^+^/0 μM Ca^2+^,145 mM Cs^+^/1 μM Ca^2+^, 0 mM Cs^+^/75 mM Ca^2+^, 145 mM Cs^+^/1 μM Ca^2+^. **E**: Quantification of macroscopic currents at –50 mV induced by ATP depletion or diazoxide in activated or non-activated (CTR) murine BEC; currents were blocked by glibenclamide and by gene deletion of *Abcc8* (SUR1KO) and gene suppression of *Trmp4* (siRNA); cells/condition for each bar: 35, 12, 14, 19, 11, 11; 24, 19; 9, 13; **, *P*<0.01; ***, *P*<0.001. **F**: Immunoblots for pCaMKII and CaMKII, with densitometric quantification of pCaMKII in murine BEC without activation, under control conditions (CT) and after exposure to the Ca^2+^ ionophore (CI), A23187 (5 μM × 10 minutes), and in activated BEC exposed to TNF in the absence and presence of glibenclamide (Glib); both bands shown had molecular masses of 50 kD, corresponding to pCaMKIIα and CaMKIIα; pCaMKII normalized to levels induced by A23187; n = 5.

The promoter regions of the genes that encode SUR1 and TRPM4, *Abcc8* and *Trmp4*, both contain canonical p65 binding elements [30,39]. To ascertain their functionality, we studied luciferase constructs of both promoters, expressed in HepG2 cells. NF-κB activation by TNF stimulated both promotors, as well as the positive control (Fig 2A). In murine BEC, NF-κB activation caused upregulation of transcripts for *Abcc8* and *Trpm4* (Fig 2B).

NF-κB activation of murine BEC upregulated cell membrane channels that exhibited the characteristic properties of the non-selective cation channel, SUR1-TRPM4 [10]: channel currents were carried by Cs^+^ [14,15], which excludes involvement of K_ATP_ channels; currents were activated by depleting intracellular ATP, increasing [Ca^2+^]_i_ or applying the SUR1 agonist, diazoxide [14,17]; and currents were inhibited by the SUR1 antagonist, glibenclamide [40], and by the cell-permeable TRPM4 antagonist, 9-phenanthrol (Fig 2C and 2E), which acts intracellularly [41] and, importantly, does not block TRPC3 [42]. SUR1-TRPM4 channels were sensitive to intracellular Ca^2+^, showed no inactivation during sustained Ca^2+^ elevations, and were not permeable to Ca^2+^ (Fig 2D). Activation failed to upregulate functional channels in endothelial cells following gene suppression of either *Abcc8*/SUR1 or *Trpm4*/TRPM4 (Fig 2E).

The normal function of the SUR1-TRPM4 channel is to act as a negative regulator of Ca^2+^ influx [43]. Accordingly, inhibition of SUR1-TRPM4 increases [Ca^2+^]_i_, an effect that can lead to an increase in pCaMKII [28]. Here also, we found an increase in pCaMKII, with no change in CaMKII, when activation of murine BEC was carried out in the presence of glibenclamide (Fig 2F; see also S1 Fig), consistent with the normal inhibitory effect of the SUR1-TRPM4 channel on Ca^2+^ influx.

### tPA opens the SUR1-TRPM4 channel

**Fig 3:**
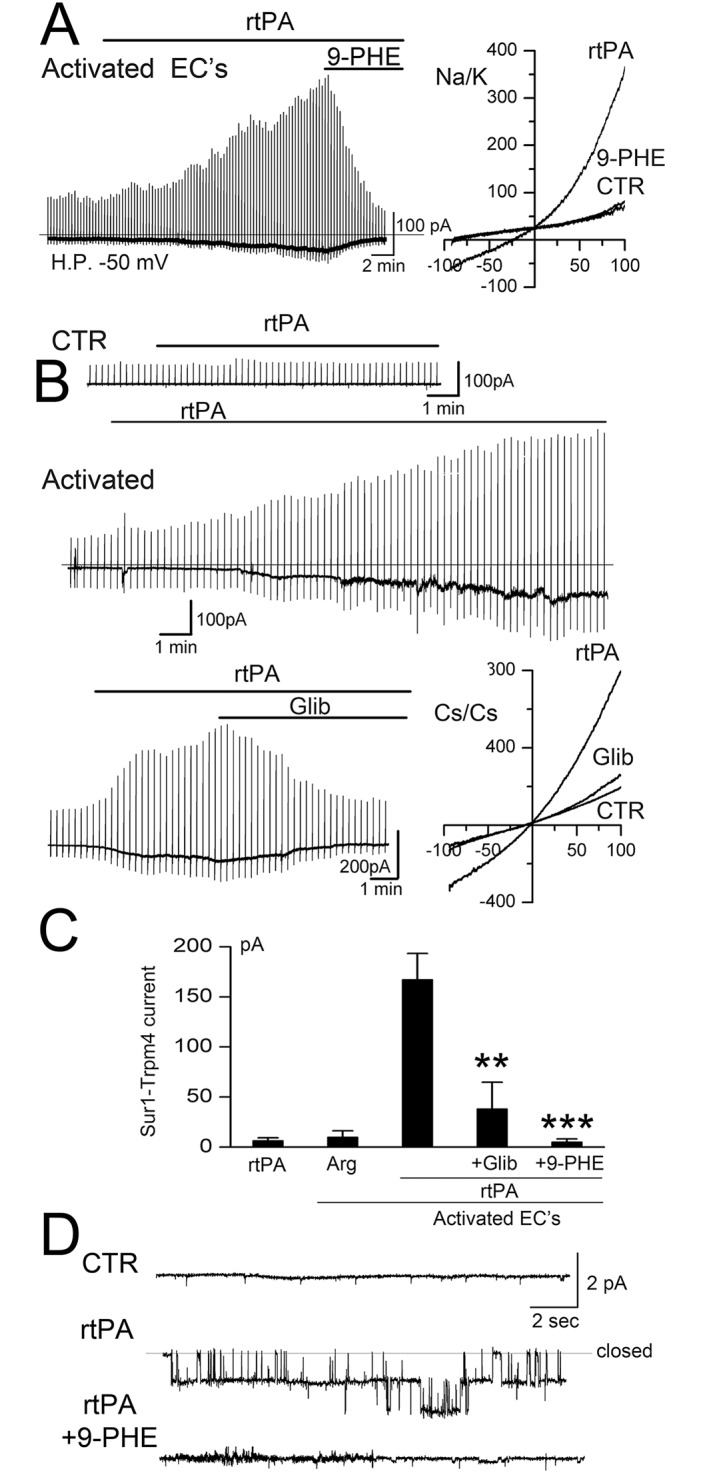
rtPA opens SUR1-TRPM4 channels. **A–C**: Macroscopic currents induced by recombinant tPA (rtPA) in activated murine BEC, recorded using physiological solutions (**A**) and using solutions with Cs^+^ as the charge carrier to block K_ATP_ (**B**); note that rtPA-induced currents could be slowly developing and long-lasting (**A,B**); currents in activated BEC were blocked by glibenclamide (Glib) and 9-phenanthrol (9-PHE); currents were not induced by rtPA in non-activated (CTR) cells or, in activated BEC, by L-arginine (Arg) (**C**); cells/condition for each bar: 16, 14, 25, 9, 5; **, *P*<0.01; ***, *P*<0.001. **D**: Single channel current, ~20 pS, in a cell-attached patch with two single channel levels before (CTR), after bath application of rtPA outside of the pipette (rtPA), and after addition of the cell-permeable TRPM4 antagonist, 9-phenanthrol (rtPA+9-PHE); holding potential, –70 mV; representative of 5 patches.

In activated murine BEC, but not in non-activated BEC, rtPA (20 μg/mL) induced both macroscopic and single channel SUR1-TRPM4 currents (Fig 3). Channel opening was observed with rtPA as low as 1–2.5 μg/mL, although these responses developed more slowly. rtPA-induced currents were observed when normal physiological salt solutions were used, as well as when Cs^+^ was used as the charge carrier to block K_ATP_ channels (Fig 3A and 3B). The rtPA-induced currents were blocked by both glibenclamide and 9-phenanthrol, consistent with SUR1-TRPM4 (Fig 3A–3C). Single channel currents of ~25 pS [10] were induced by rtPA when cells were studied using a cell-attached configuration (Fig 3D), with this specific configuration used to preclude a direct effect of rtPA on the extracellular face of channel itself. Control experiments showed that SUR1-TRPM4 channel opening was not due to L-arginine, which is present in rtPA (Alteplase^®^) (Fig 3C).

It was previously shown that outward rectification of TRPM4 is observed with PIP_2_ depletion [44,45]. Of note, macroscopic SUR1-TRPM4 currents induced by rtPA exhibited outward rectification (Fig 3A and 3B), whereas macroscopic SUR1-TRPM4 currents induced by ATP depletion (Fig 2C) or by the calcium ionophore A23187 (*vide infra*) exhibited no rectification. Outward rectification with rtPA is consistent with rtPA (but not ATP depletion or A23187) causing PIP_2_ depletion due to GPCR activation.

### SUR1-TRPM4 channel opening by tPA requires Ca^2+^ influx—Possible involvement of TRPC3

**Fig 4:**
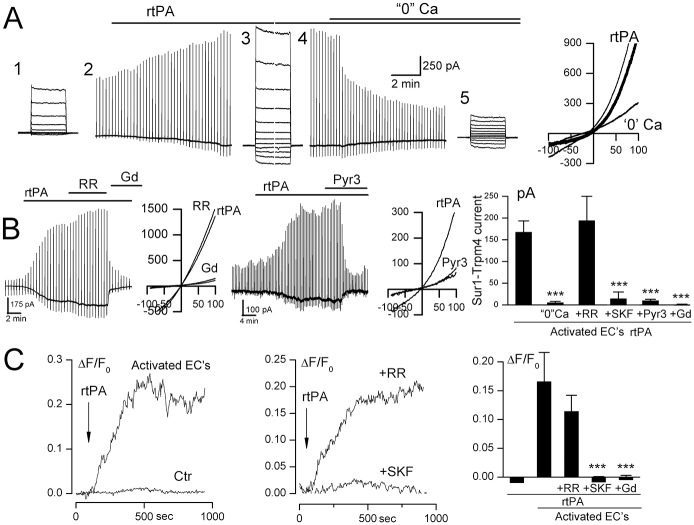
SUR1-TRPM4 channel opening by tPA requires Ca^2+^ influx via TRPC3. **A**: Macroscopic currents during 200-ms step pulses (#1,3,5) and ramp pulses (–100 to +100 mV; 4 mV/msec) (#2,4), induced by recombinant tPA (rtPA) in activated murine BEC, recorded initially using extracellular solution with 1.8 mM Ca^2+^, and after switching to extracellular solution containing 0 mM Ca^2+^; *right*: illustrative currents during ramp pulses after addition of rtPA, before and after switch to 0 Ca^2+^; the difference current is also shown (thick line). **B**: rtPA-induced current in activated BEC was not blocked by ruthenium red (RR), but was blocked by Gd^+3^ and Pyr3; illustrative currents during ramp pulses after addition of rtPA, before and after Gd^+3^ or Pyr3 are also shown; *bar graph*: rtPA-induced currents in activated BEC in the presence of 1.8 mM Ca^2+^, 0 mM Ca^2+^; 1.8 mM Ca^2+^ plus RR or SKF-96365 or Pyr3 or Gd^3+^; cells/condition for each bar: 25, 7, 5, 7, 5, 7. **C**: Change in intracellular Ca^2+^ concentration (ΔF/F_0_) induced by rtPA in activated but not in non-activated (Ctr) BEC; the rtPA-induced increase in Ca^2+^ was blocked by pretreatment with SKF-96365 but not RR; *bar graph*: mean change at 10–12 minutes in intracellular Ca^2+^ concentration induced by rtPA in non-activated and activated BEC, in the presence of RR; SKF-96365; Gd^3+^; cells/condition for each bar: 10, 8, 10, 10, 10; ***, *P*<0.001.

We hypothesized that SUR1-TRPM4 channels, which are known to be opened by an increase in [Ca^2+^]_i_ [43], were being opened by Ca^2+^ influx induced by rtPA. The removal of extracellular Ca^2+^ resulted in a gradual loss of SUR1-TRPM4 channel activity (Fig 4A and 4B), consistent with a requirement for Ca^2+^ influx to maintain SUR1-TRPM4 channel opening. Step pulses during rtPA application showed that SUR1-TRPM4 currents were sustained at hyperpolarizing potentials (Fig 4A-3), consistent with sustained, not transient, increases in intracellular Ca^2+^ induced by rtPA. Gd^3+^, a non-specific blocker of Ca^2+^-permeable channels that also blocks TRPM4 [46,47], reduced SUR1-TRPM4 channel activity (Fig 4B).

We examined the mechanism of Ca^2+^ influx. Channel activity was not affected by the TRPV channel blocker, ruthenium red, but was strongly reduced by SKF-96365 and Pyr3 (Fig 4B), with Pyr3 being an established inhibitor of TRPC3 [48] that also inhibits Orai1 [49].

We performed Ca^2+^ imaging experiments to confirm that rtPA caused Ca^2+^ influx. rtPA induced a sustained rise in intracellular Ca^2+^ in activated BEC, but not in non-activated BEC (Fig 4C). As with SUR1-TRPM4 channel activity, Ca^2+^ influx was blocked by Gd^3+^ and SKF-96365, but not by ruthenium red (Fig 4C) [50].

Our Ca^2+^ imaging experiments invariably demonstrated that rtPA induced a slowly developing rise in intracellular Ca^2+^ that was sustained, not transient. Similarly, our patch clamp experiments demonstrated that rtPA induced SUR1-TRPM4 currents that were sustained during step pulses (Fig 4A-3), consistent with sustained Ca^2+^ influx. Although, as noted, SKF-96365 and Pyr3 can inhibit both TRPC3 and Orai1, Orai1 characteristically gives rise to an inactivating or partially inactivating Ca^2+^ influx [51], with inactivation due to Ca^2+^-induced phosphorylation of STIM1 [52]. By contrast, GPCR-mediated TRPC3 activation characteristically gives rise a slowly developing, sustained Ca^2+^ influx [53,54]. Thus, Pyr3-sensitive, non-inactivating Ca^2+^ influx induced by rtPA likely was due to TRPC3, not Orai1, with TRPC3-mediated Ca^2+^ influx resulting in SUR1-TRPM4 channel activation.

### Proteolytic activity of tPA is required

**Fig 5:**
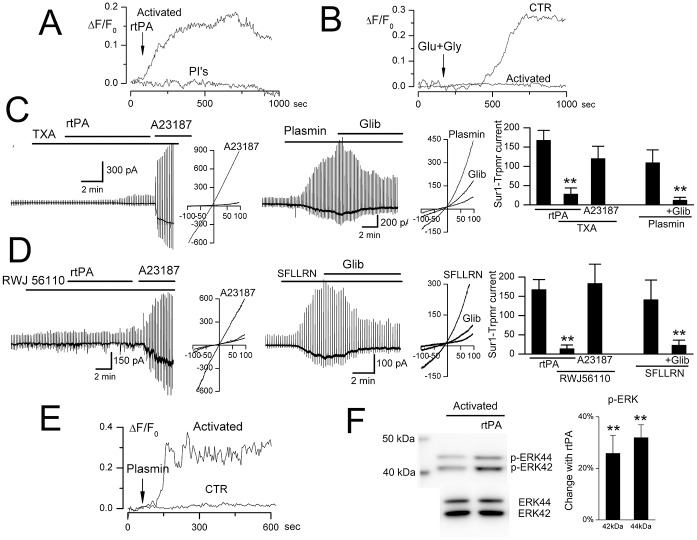
Plasmin opens the SUR1-TRPM4 channel. **A**: Control experiment showing requirement for proteolytic activity; change in intracellular Ca^2+^ concentration (ΔF/F_0_) induced by recombinant tPA (rtPA) in activated murine BEC, in the absence and presence of the non-specific serine threonine protease inhibitors (PI’s), aprotinin and leupeptin; representative of 5–7 cells per condition. **B**: Control experiment showing non-involvement of NMDA (N-methyl-D-aspartate) receptor; change in intracellular Ca^2+^ induced by glutamate plus glycine (Glu+Gly) in non-activated (CTR) and in activated BEC; representative of 7 cells per condition. **C**: In activated murine BEC, rtPA fails to induce SUR1-TRPM4 current in the presence of tranexamic acid (TXA), although Ca^2+^ influx via A23187 activates the channel; exogenous plasmin induces SUR1-TRPM4 current blocked by glibenclamide (Glib); bar graph showing SUR1-TRPM4 currents under the conditions indicated; illustrative currents during ramp pulses are also shown; cells/condition for each bar: 25, 11, 8, 5, 5. **D**: In activated murine BEC, rtPA fails to induce SUR1-TRPM4 current in the presence of PAR1-antagonist RWJ56110, although Ca^2+^ influx via A23187 activates the channel; PAR1-agonist SFLLRN induces SUR1-TRPM4 current that is blocked by glibenclamide; bar graph showing SUR1-TRPM4 currents under the conditions indicated; illustrative currents during ramp pulses are also shown; cells/condition for each bar: 25, 12, 11, 5, 5. **E**: Plasmin induces Ca^2+^ influx (ΔF/F_0_) in activated but not non-activated (CTR) murine BEC; 5–8 cells per condition. **F**: rtPA induces phosphorylation of ERK1/2 (p42/44 MAPK); bar graph showing >20% increase in p-ERK42 and p-ERK44 due to rtPA; n = 3; **, *P*<0.01.

The non-specific serine threonine protease inhibitors, aprotinin and leupeptin, prevented rtPA-induced Ca^2+^ influx in activated BEC (Fig 5A), indicating a requirement for proteolysis by rtPA.

Proteolytic cleavage of N-methyl-D-aspartate receptor (NMDAR) by rtPA has been shown to augment Ca^2+^ influx in neurons [55]. In non-activated BEC, Ca^2+^ influx was induced by glutamate, but this effect was not augmented by rtPA, and glutamate-induced Ca^2+^ influx was lost after BEC activation (Fig 5B). These data indicate that NMDAR is not the proteolytic target of rtPA in activated BEC.

We excluded other potential targets of proteolysis. In COS-7 cells that stably express TRPM4 (but not PAR1 [56] or TLR4 [57]), TRPM4 channels could be activated by the Ca^2+^ ionophore, A23187, but not by rtPA (not shown). As noted above, opening of single SUR1-TRPM4 channels in cell-attached patches precludes proteolytic action on either subunit of the channel, since the channels are shielded by the pipette, suggesting that SUR1 and TRPM4 were not the proteolytic target of rtPA.

### SUR1-TRPM4 channel opening by tPA requires plasmin

Plasminogen binds to endothelial cells [58], bound plasminogen can be activated by tPA [58], and cell surface-generated plasmin can activate PAR1 or TLR4 [9], both of which can induce Ca^2+^ influx via DAG-sensitive TRPC3/6 [59]. Consistent with involvement of plasmin, tranexamic acid, which competitively inhibits the activation of plasminogen to plasmin, prevented the induction of SUR1-TRPM4 channel currents by rtPA in activated BEC, although currents still could be induced by the Ca^2+^ ionophore, A23187 (Fig 5C). Also, exogenous plasmin recapitulated the effects of rtPA on activated BEC, including the induction of glibenclamide-sensitive SUR1-TRPM4 currents and the induction of Ca^2+^ influx (Fig 5C and 5E).

### SUR1-TRPM4 channel opening by tPA requires PAR1

Consistent with involvement of PAR1, the PAR1-selective antagonist, RWJ56110 [60], prevented the induction of SUR1-TRPM4 channel currents by rtPA in activated BEC, although currents still could be induced by the Ca^2+^ ionophore, A23187 (Fig 5D). Also, application of the PAR1-agonist peptide, SFLLRN, recapitulated the effect of rtPA on activated BEC [61], causing the induction of glibenclamide-sensitive SUR1-TRPM4 channel currents (Fig 5D).

Canonical activation of PAR1 by cleavage of the N-terminus exodomain at Arg41 results in activation of the MAPK pathway, which can be demonstrated by rapid phosphorylation of ERK1/2 (p42/44 MAPK) at Thr202/Tyr204 [6,62]. ERK1/2 phosphorylation was increased by activation alone, and was further increased by brief exposure to rtPA (Fig 5F; see also S2 Fig), consistent with canonical Arg41-PAR1 signaling induced by rtPA.

Apart from PAR1, plasmin also can signal via TLR4 [9], and TLR4 can activate DAG-sensitive Trpc channels [63]. Potential involvement of TLR4 was excluded, however, as plasmin-induced Ca^2+^ influx was not blocked by the specific TLR4 antagonist, TAK-242 (S3 Fig).

Together, these findings demonstrated involvement of the plasmin/PAR1/Trpc/Ca^2+^ pathway in SUR1-TRPM4 channel opening by rtPA in activated BEC.

### Tonic vs. phasic MMP-9 secretion

ProMMPs, which require proteolytic cleavage to become mature proteases, are secreted via two distinct mechanisms: (i) MMPs undergo *tonic* secretion via the normal secretory pathway [23]; (ii) MMPs undergo *phasic* secretion following GPCR activation [24]. Notably, receptor-induced secretion requires Ca^2+^ influx [25].

**Fig 6:**
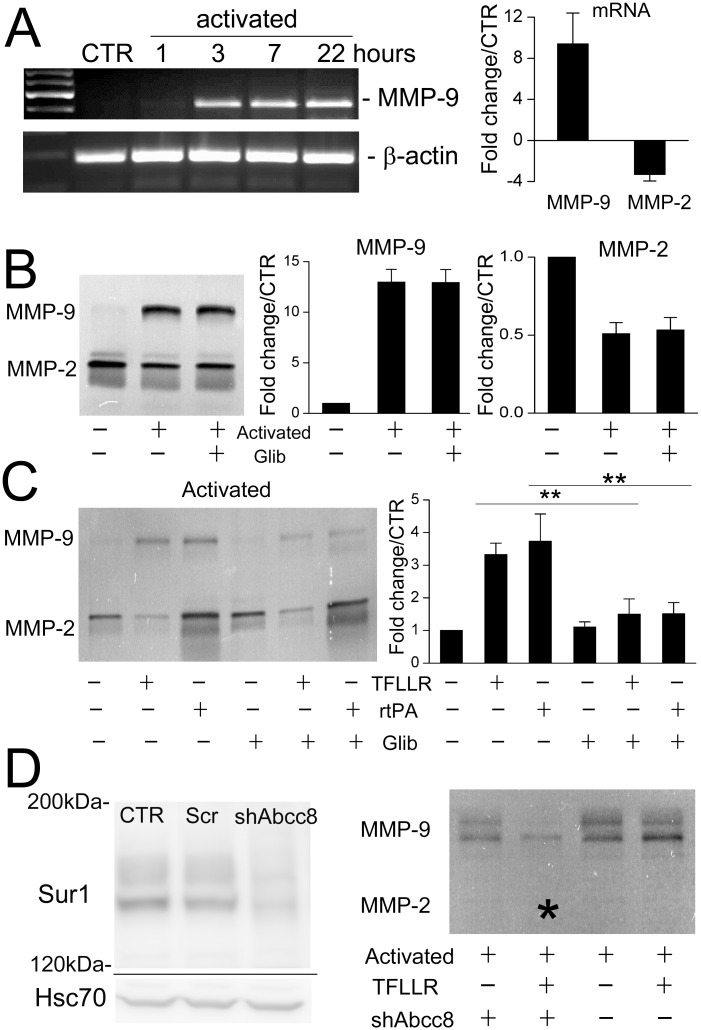
rtPA and PAR1-agonist cause secretion of MMP-9 from activated endothelial cells that is reduced by SUR1 inhibition. **A**: RT-PCR showing that activation of murine BEC upregulates MMP-9 but not MMP-2 mRNA; n = 5. **B**: Gelatin zymography showing that non-activated murine BEC secrete minimal MMP-9, and that *tonic* secretion of MMP-9 and MMP-2, both pro and active forms, following overnight activation is not affected by glibenclamide; n = 5. **C**: Gelatin zymography showing that after activation, rtPA and PAR1-agonist TFLLR induce *phasic* secretion of MMP-9 that is inhibited by glibenclamide; n = 5; **, *P*<0.01. **D** *left*: Immunoblot showing suppression of SUR1 after infection with shAbcc8 lentiviral vector; CTR, untreated cells; Scr, lentiviral vector with scrambled shRNA. **D** *right*: Gelatin zymography showing that after overnight activation, *phasic* secretion of MMP-9 induced by TFLLR is inhibited by pretreatment of the cells with shRNA against *Abcc8* (*); n = 3.

In non-activated BEC, MMP-9 was minimally expressed (Fig 6A and 6B). However, activation caused a several-fold upregulation of MMP-9 mRNA, and a significant increase in gelatinase activity, consistent with *tonic* secretion of MMP-9 (Fig 6A and 6B). MMP-2 mRNA and gelatinase activity were slightly decreased (Fig 6A and 6B) [64].

In addition to tonic secretion, *phasic* secretion of MMP-9 by endothelial cells can be triggered by receptor activation [24]. Both rtPA and the PAR1-agonist peptide, TFLLR, induced a marked increase in MMP-9 gelatinase activity (Fig 6C). Activation was required, as no gelatinase activity was released by rtPA or TFLLR applied to non-activated BEC (not shown).

### SUR1 inhibition and tonic vs. phasic MMP-9 secretion

The addition of glibenclamide during activation of BEC had no effect on tonic MMP-9 secretion (Fig 6B). However, glibenclamide significantly reduced phasic MMP-9 secretion induced by rtPA (Fig 6C). This last finding was unexpected, since Ca^2+^ influx is required for phasic MMP-9 secretion induced by GPCR activation [65], and SUR1-TRPM4 inhibition *increases* intracellular Ca^2+^ (*see* Fig 2E). A similar inhibitory effect of glibenclamide on phasic secretion of MMP-9 was observed with TFLLR-activation of PAR1 (Fig 6C). Importantly, previous work had shown that glibenclamide does not affect the proteolytic activity of rtPA [20].

The effect of glibenclamide was reproduced in activated BEC expressing shRNA directed against *Abcc8* (Fig 6D), confirming that the effect of glibenclamide was due to SUR1 inhibition, and showing again that Ca^2+^ dysregulation produced by inhibiting SUR1-TRPM4 channel strongly reduced phasic MMP-9 secretion.

### Human BEC

**Fig 7:**
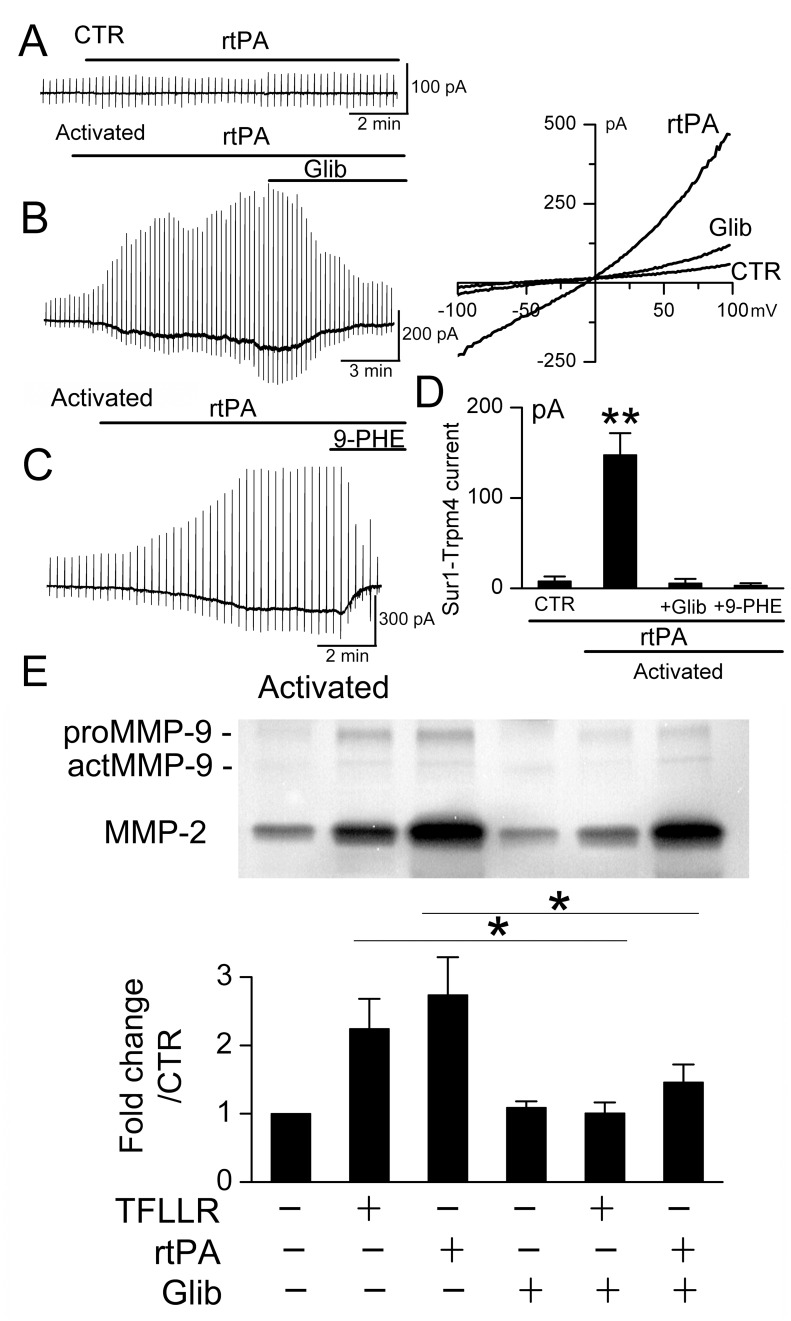
Human BEC upregulate SUR1-TRPM4 channels that are involved in tPA-induced phasic secretion of MMP-9. **A–D**: Macroscopic currents induced by recombinant tPA (rtPA) in activated but not in non-activated (CTR) human BEC; currents were blocked by glibenclamide (Glib) and 9-phenanthrol (9-PHE); n = 6–10 cells/condition; **, *P*<0.01. **E**: Gelatin zymography showing that after activation, rtPA and PAR1-agonist TFLLR induce *phasic* secretion of MMP-9 in human BEC that is inhibited by glibenclamide; the bar graphs represent densitometric measurements of total MMP-9; the lanes and the bars in the graph are aligned for the different conditions: PAR1-agonist (TFLLR), rtPA and glibenclamide (Glib); n = 4; *, *P*<0.05.

The foregoing experiments were performed using murine BEC. To examine the potential clinical significance of SUR1 inhibition on MMP-9 secretion, we replicated key experiments using human BEC. In these cells as well, activation resulted in upregulation of SUR1-TRPM4 channels that could be activated by rtPA (Fig 7A–7D). As with murine BEC, tonic secretion of MMP-9 by activated human BEC was not influenced by SUR1 inhibition (not shown), whereas phasic secretion of MMP-9 induced by rtPA or by the PAR1-agonist peptide, TFLLR, was significantly reduced by glibenclamide (Fig 7E). Unlike murine BEC, expression of MMP-2 was prominent in human BEC, and phasic secretion of MMP-2 was slightly reduced by glibenclamide (Fig 7E, *upper panel*).

## Discussion

**Fig 8:**
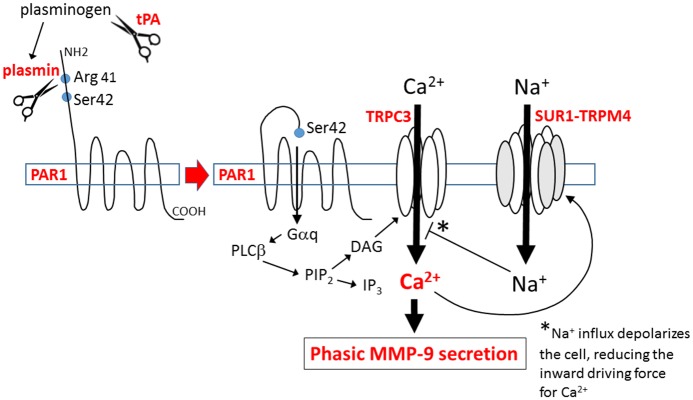
Proposed mechanism for tPA-induced phasic secretion of MMP-9 by NF-κB-activated brain endothelium. tPA catalyzes the cleavage of plasminogen, yielding plasmin. Plasmin activates the G-protein coupled receptor, PAR1, by proteolytic cleavage of its N-terminal domain at Arg 41, allowing its tethered ligand to bind intramolecularly to activate the receptor. Activated PAR1 signals via the G-protein, Gαq, activating phospholipase Cβ (PLCβ), which catalyzes the cleavage of membrane-bound phosphatidylinositol 4,5-biphosphate (PIP_2_) into the second messengers, inositol (1,4,5) trisphosphate (IP_3_) and diacylglycerol (DAG). DAG activates TRPC3, resulting in Ca^2+^ influx. DAG-induced Ca^2+^ influx triggers phasic secretion of MMP-9, and causes activation of SUR1-TRPM4, which results in Na^+^ influx. Cell depolarization due to Na^+^ influx reduces the inward electrochemical driving force for Ca^2+^, consistent with SUR1-TRPM4 functioning as a negative regulator of Ca^2+^ influx.

Here, we demonstrated that tPA induces MMP-9 secretion from activated brain endothelial cells, and we elucidated the signaling mechanism involved. We found that NF-κB-activation of BEC, as occurs *in vivo* during I/R, caused *de novo* expression of SUR1-TRPM4 channels and, in activated BEC: (i) tPA opens SUR1-TRPM4 channels; (ii) SUR1-TRPM4 channel activity is involved in the phasic secretion of MMP-9 induced by tPA; (iii) both the activation of SUR1-TRPM4 channels and the phasic secretion of MMP-9 require PAR1 signaling. Key aspects of the findings made in murine BEC were replicated in human BEC, lending important translational relevance to our studies. Notably, the signaling cascade identified here involving tPA/plasmin, PAR-1 and Ca^2+^ in MMP-9 secretion in NF-κB-activated brain endothelial cells (Fig 8) is similar to the cascade involving thrombin, PAR-1 and Ca^2+^ in MMP-9 secretion in pericytes and NF-κB-activated monocytes [66,67].

The present study is the first to show that native SUR1-TRPM4 channels are opened by Ca^2+^ influx induced by G-protein coupled receptor activation. Previously, SUR1-TRPM4 channel opening in cerebral ischemia was attributed exclusively to pathological depletion of ATP [43]. Linking the SUR1-TRPM4 channel to PAR1 signaling broadens the pathological implications of channel upregulation, since in ischemia, endogenous tPA/plasmin as well as thrombin both signal via PAR1, and may be responsible for receptor-induced MMP-9 secretion in the absence of exogenous (recombinant) tPA [68].

In cerebral ischemia, neutrophils are believed to comprise a critical source of MMP-9 responsible for hemorrhagic transformation [69], but other cell types intrinsic to the brain, including neurons, microglia, pericytes and endothelial cells also express MMP-9 (see review [69]). During the first 24 hours after focal ischemia, MMP-9 appears predominantly within microvascular endothelial cells and neutrophils, both within and at the periphery of the infarct [37], with laser capture microscopy clearly demonstrating that the microvascular endothelium in ischemic areas contains high levels of MMP-9 [38]. Thus, the study of microvascular endothelium adds an important dimension to our understanding of MMP biology in the brain following I/R.

*In vitro* models of stroke frequently mimic cerebral ischemia using oxygen-glucose deprivation (OGD). However, OGD does not fully recapitulate the pathological effects of I/R, specifically, the activation of NF-κB signaling. With *in vivo* MCAo models, one of the hallmarks of I/R is activation of NF-κB as early as 15 minutes after reperfusion [34,36], an event that is later accompanied by upregulation of TNF [64]. Blockage of either TNF [64] or NF-κB [34,35] ameliorates the effects of I/R and reduces MMP-9 levels [64]. Moreover, *in vitro*, when human BEC are subjected to hypoxia followed by reoxygenation, but not hypoxia alone, an NF-κB complex composed of p65 and p50 Rel proteins is rapidly activated within 15–30 minutes [70]. In human BEC, a robust increase in MMP-9 protein is obtained by TNF-mediated NF-κB activation, but not by OGD [71]. Thus, the study of BEC following NF-κB activation further broadens our understanding of MMP biology in the brain following I/R.

PAR1 plays an important role in cerebral ischemia [72]. Canonical cleavage by thrombin and plasmin at Arg 41 stabilizes a PAR1 conformation that preferentially associates with G-proteins of the Gq and G12/13 families [73], induces ERK1/2 phosphorylation [62], and leads to innate inflammation and BBB disruption. Here, we confirmed that tPA leads to canonical PAR1 signaling. In cerebral ischemia, silencing PAR1 reduces neurological deficits, reduces BBB leakage, and decreases neuronal degeneration [74], but PAR1 inhibitors also increase hemorrhagic transformation [75]. The work presented here and previously [19–22] demonstrates that inhibiting SUR1-TRPM4 signaling downstream of canonical PAR1 activation can preserve the benefit of PAR1 inhibition without incurring the undesirable pro-hemorrhagic effect.

Hemorrhagic transformation results from the catastrophic structural failure of the microvasculature [76]. In stroke, hemorrhagic transformation occurs predominantly in regions subjected to I/R, not usually in normal brain or, logically, in tissues that are not perfused. Thus, it is primarily endothelium that has been activated by I/R, *i*.*e*., endothelium that expresses SUR1-TRPM4, that it is at risk from tPA. Also, rtPA administration in stroke patients is generally considered to be less safe at later times after onset of ischemia, reflecting, perhaps, the time required for transcriptional upregulation of SUR1-TRPM4 channels [13]. These observations, coupled with the findings reported here, are consistent with the notion that the PAR1/TRPC3/SUR1-TRPM4 axis in activated BEC is a target of tPA in cerebral ischemia. Most important, our findings are consistent with the preclinical and clinical data showing that sulfonylurea antagonists reduce plasma MMP-9 levels and hemorrhagic transformation in cerebral ischemia [19–22]. Also, we found that SUR1 inhibition reduced receptor-mediated *phasic* secretion but not *tonic* secretion of MMP-9 from activated endothelium. This observation predicts that sulfonylurea antagonists will reduce levels of MMP-9 only partially, but not completely, precisely as found in human stroke [21,22]. Overall, our findings support the concept of an important role for a plasmin-linked mechanism in MMP secretion and hemorrhagic transformation, which may supplement plasminogen/plasmin-independent mechanisms [4,5].

The precise mechanism by which SUR1 blockade inhibits GPCR-mediated phasic secretion of MMP remains to be determined. Our findings led to the apparent paradox that, on the one hand, Ca^2+^ influx is required for GPCR-induced phasic secretion of MMP from activated endothelial cells [25] whereas, on the other hand, SUR1 blockade, which increases intracellular Ca^2+^ [43], inhibits phasic secretion of MMP. Disrupting intracellular Ca^2+^ homeostasis by inhibiting SUR1-TRPM4 can affect numerous Ca^2+^-dependent factors and pathways, and thus several mechanisms potentially could be involved. One that we favor involves desensitization and internalization of PAR1, enhanced by elevated levels of pCaMKII, a mechanism that would specifically target phasic but not tonic secretion. GPCR activation is known to trigger receptor desensitization and internalization that is dependent upon GPCR kinase, arrestin and clathrin, as well as CaMKII [77,78], the latter implicated in phosphorylation of the receptor [79,80] and of arrestin [81]. Consistent with this, our data showed that SUR1-TRPM4 inhibition resulted in a significant increase in pCaMKII (Fig 2E). Further work will be required to clarify whether pCaMKII augments PAR1 desensitization in BEC.

## Conclusions

tPA induces PAR1-mediated, Ca^2+^-dependent phasic secretion of MMP-9 from activated brain endothelium, and SUR1-TRPM4 is required for this process. To the extent that MMP-9 secretion is essential for hemorrhagic transformation, the data presented here are important for understanding mechanisms of hemorrhagic transformation in stroke. Emerging evidence indicates that glibenclamide exerts important beneficial effects in cerebral ischemia, especially in humans with large hemispheric infarctions [21,22], reaffirming a critical role for SUR1-TRPM4 channels in stroke.

## Supporting information

S1 Fig Immunoblot of CaMKII. Representative immunoblots for pCaMKII (*left*) and CaMKII (*right*) from bEnd.3 cells at different times after treatment with Ca^2+^ ionophore (A23187) or TNF (20 ng/mL), without and with glibenclamide.
(TIF)

S2 Fig Immunoblot of ERK. Representative immunoblot for ERK1/2 and pERK1/2 from activated bEnd.3 cells in control (CTR) and after 10-minute exposure to rtPA.
(TIF)

S3 Fig TAK-242 does not inhibit the effect rtPA on calcium influx. Change in intracellular Ca^2+^ concentration (ΔF/F_0_) induced by rtPA in activated BEC; the rtPA-induced increase in Ca^2+^ was not blocked by pretreatment with TAK-242; *bar graph*: mean change at 10–12 minutes in intracellular Ca^2+^ concentration induced by rtPA in activated BEC in the presence of TAK-242; 7 cells.
(TIF)

S1 File Original data. This file contains the original data quantified in the figures.
(XLSX)
