# Supplementary figures and images for: SUR1-TRPM4 channel activation and phasic secretion of MMP-9 induced by tPA in brain endothelial cells
Source: PLoS One. 2018 Apr 4;13(4):e0195526. doi: 10.1371/journal.pone.0195526 (PMC5884564; doi:10.1371/journal.pone.0195526)

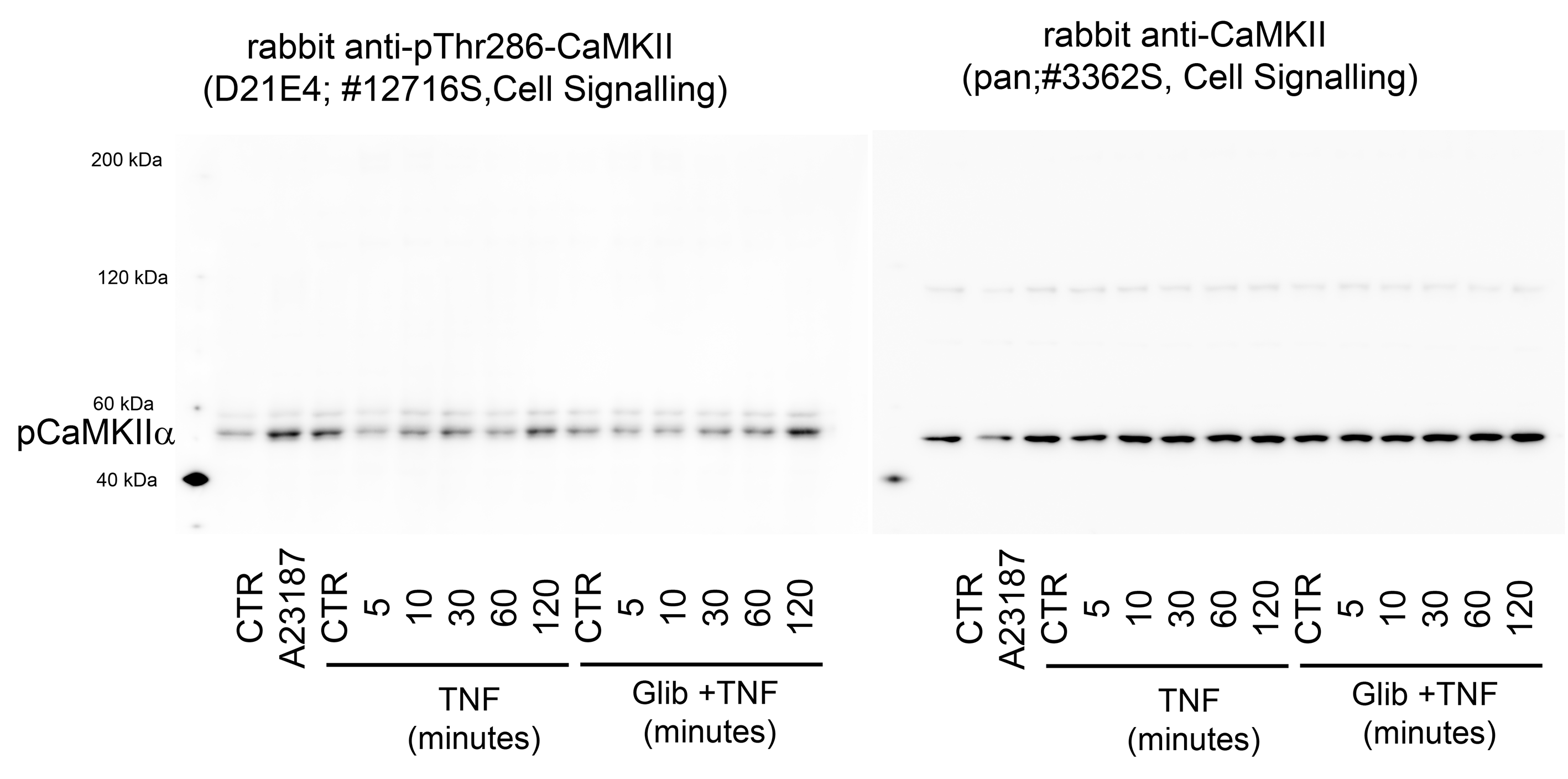

Supplement: S1 Fig — Representative immunoblots for pCaMKII (left) and CaMKII (right) from bEnd.3 cells at different times after treatment with Ca2+ ionophore (A23187) or TNF (20 ng/mL), without and with glibenclamide. (TIF) [file pone.0195526.s001.tif]

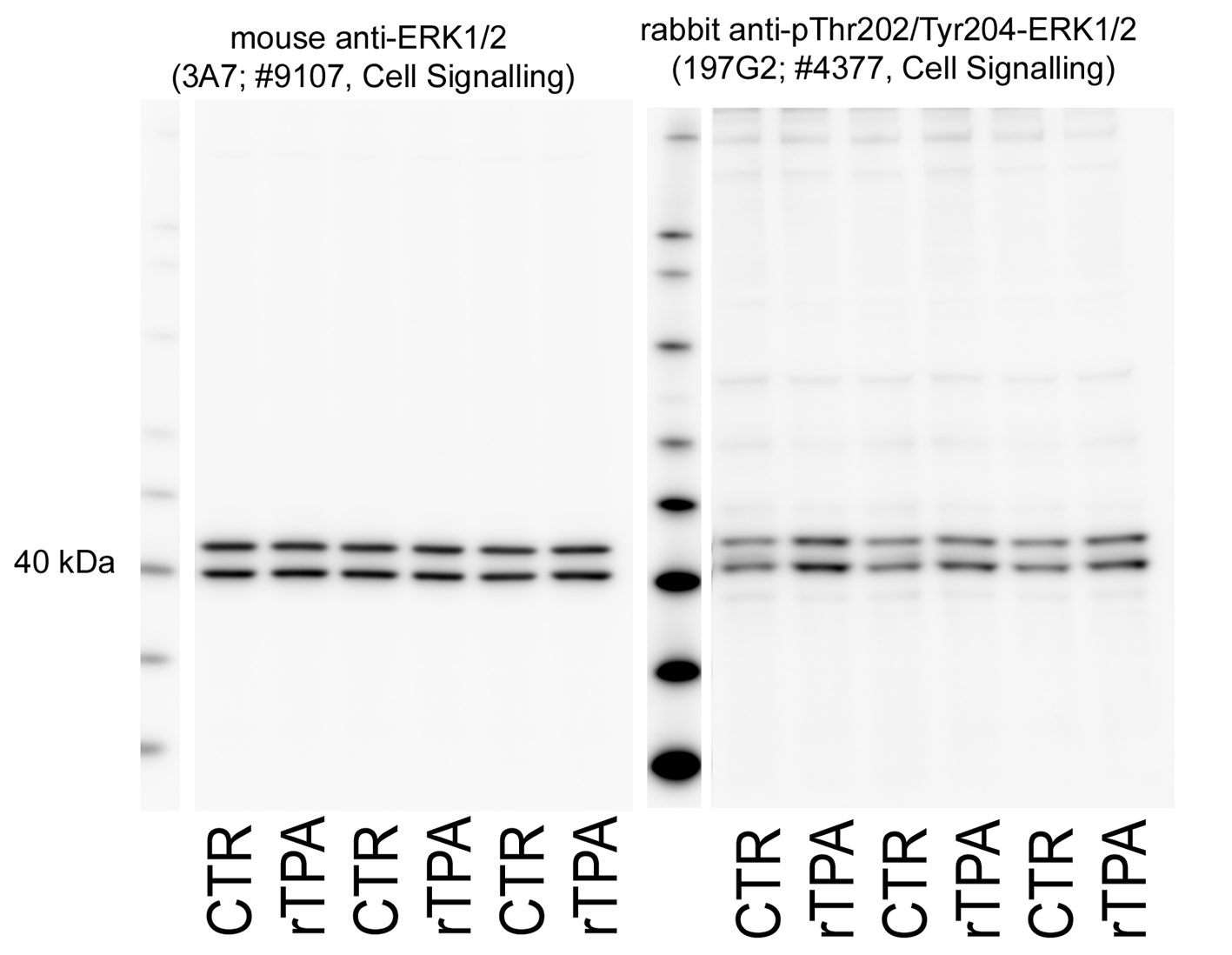

Supplement: S2 Fig — Representative immunoblot for ERK1/2 and pERK1/2 from activated bEnd.3 cells in control (CTR) and after 10-minute exposure to rtPA. (TIF) [file pone.0195526.s002.tif]

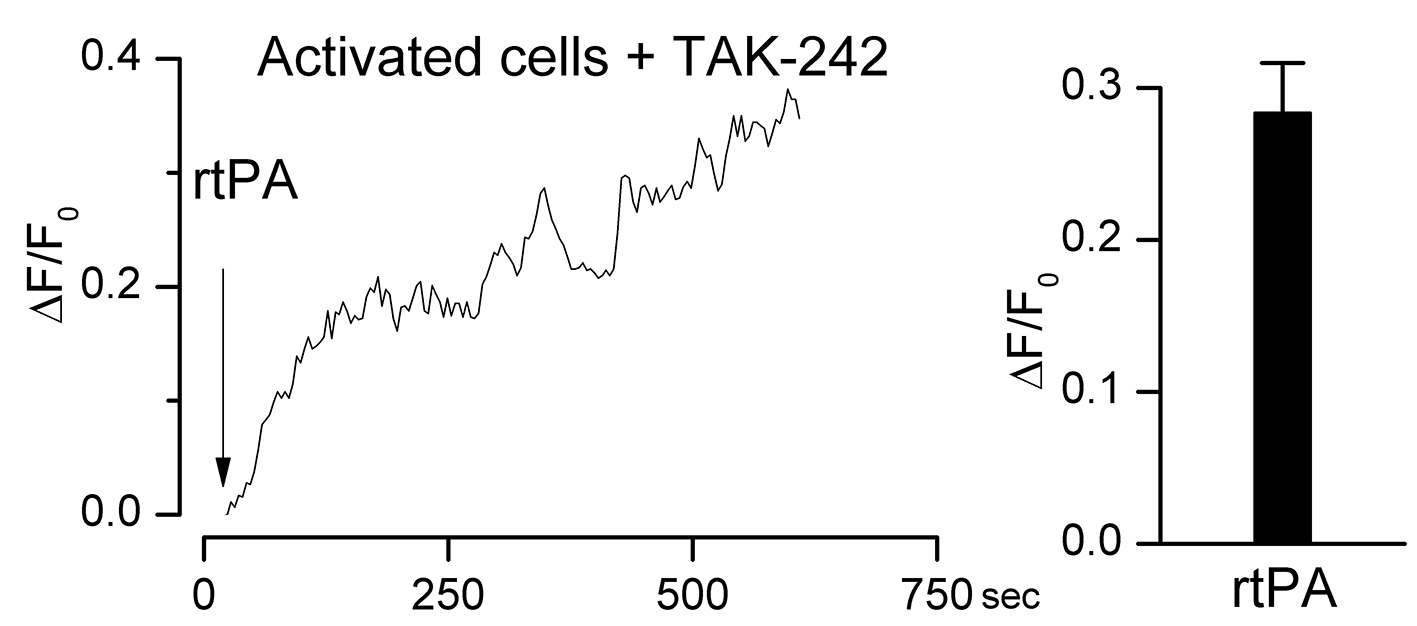

Supplement: S3 Fig — Change in intracellular Ca2+ concentration (ΔF/F0) induced by rtPA in activated BEC; the rtPA-induced increase in Ca2+ was not blocked by pretreatment with TAK-242; bar graph: mean change at 10–12 minutes in intracellular Ca2+ concentration induced by rtPA in activated BEC in the presence of TAK-242; 7 cells. (TIF) [file pone.0195526.s003.tif]
